# Success stories and emerging themes in conservation physiology

**DOI:** 10.1093/conphys/cov057

**Published:** 2016-01-05

**Authors:** Christine L. Madliger, Steven J. Cooke, Erica J. Crespi, Jennifer L. Funk, Kevin R. Hultine, Kathleen E. Hunt, Jason R. Rohr, Brent J. Sinclair, Cory D. Suski, Craig K. R. Willis, Oliver P. Love

**Affiliations:** 1 Department of Biological Sciences, University of Windsor, Windsor, ON, Canada N9B 3P4; 2 Fish Ecology and Conservation Physiology Laboratory, Department of Biology and Institute of Environmental Science, Carleton University, Ottawa, ON, Canada K1S 5B6; 3 School of Biological Sciences, Washington State University, Pullman, WA 99164, USA; 4 Schmid College of Science and Technology, Chapman University, Orange, CA 92866, USA; 5 Department of Research, Conservation and Collections, Desert Botanical Garden, Phoenix, AZ 85008, USA; 6 John H. Prescott Marine Laboratory, Research Department, New England Aquarium, Boston, MA 02110, USA; 7 Integrative Biology, University of South Florida, Tampa, FL 33620, USA; 8 Department of Biology, Western University, London, ON, Canada N6A 5B7; 9 Department of Natural Resources and Environmental Sciences, University of Illinois at Urbana-Champaign, Urbana, IL 61801, USA; 10 Department of Biology and Centre for Forest Interdisciplinary Research, University of Winnipeg, Winnipeg, MB, Canada R3B 2E9; 11 Great Lakes Institute for Environmental Research, University of Windsor, Windsor, ON, Canada N9B 3P4

**Keywords:** Conservation physiology, ecotourism, invasive species, nutrition, sensory ecology, toxicology

## Abstract

The potential benefits of physiology for conservation are well established and include greater specificity of management techniques, determination of cause–effect relationships, increased sensitivity of health and disturbance monitoring and greater capacity for predicting future change. While descriptions of the specific avenues in which conservation and physiology can be integrated are readily available and important to the continuing expansion of the discipline of ‘conservation physiology’, to date there has been no assessment of how the field has specifically contributed to conservation success. However, the goal of conservation physiology is to foster conservation solutions and it is therefore important to assess whether physiological approaches contribute to downstream conservation outcomes and management decisions. Here, we present eight areas of conservation concern, ranging from chemical contamination to invasive species to ecotourism, where physiological approaches have led to beneficial changes in human behaviour, management or policy. We also discuss the shared characteristics of these successes, identifying emerging themes in the discipline. Specifically, we conclude that conservation physiology: (i) goes beyond documenting change to provide solutions; (ii) offers a diversity of physiological metrics beyond glucocorticoids (stress hormones); (iii) includes approaches that are transferable among species, locations and times; (iv) simultaneously allows for human use and benefits to wildlife; and (v) is characterized by successes that can be difficult to find in the primary literature. Overall, we submit that the field of conservation physiology has a strong foundation of achievements characterized by a diversity of conservation issues, taxa, physiological traits, ecosystem types and spatial scales. We hope that these concrete successes will encourage the continued evolution and use of physiological tools within conservation-based research and management plans.

## Abstract (precis)

The potential benefits of a physiological approach to conservation are well-established. Here we present a cross-section of conservation physiology success stories and a discussion of their shared characteristics to illustrate how the discipline has tangibly contributed to conservation and management across a diversity of topics, taxa, and spatial scales.

## Introduction

Although the discipline of conservation physiology was formally defined only recently (Wikelski and Cooke, 2006), physiology has permeated conservation biology for decades (reviewed by Cooke *et al.*, 2013). The mechanistic approach espoused by conservation physiologists is considered powerful because it allows for the determination of cause–effect relationships (Carey, 2005; Wikelski and Cooke, 2006), contributing a valuable, evidence-based approach to conservation (Sutherland *et al.*, 2004). More specifically, the discipline integrates functional and mechanistic responses at all scales (Cooke *et al.*, 2013), leveraging diverse techniques from genomics and immunology to energetics and sensory physiology with the goal of fostering conservation solutions (Carey, 2005; Tracy *et al.*, 2006; Wikelski and Cooke, 2006; Cooke and O'Connor, 2010; Seebacher and Franklin, 2012; Cooke *et al.*, 2013). As a synergistic union of two disciplines, conservation physiology has the potential to lead to diverse tools and new theoretical paradigms (Coristine *et al.*, 2014); however, it must also contend with the differing perceptions, knowledge bases and logistical constraints from each independent discipline (Cooke and O'Connor, 2010) that may inhibit full integration (Lennox and Cooke, 2014). Importantly, successful integration of the disciplines is a multistep process that links ecological context and variation in physiology to the fitness parameters that drive population persistence (Coristine *et al.*, 2014). To have a tangible conservation impact, this information must then be translated into management recommendations, recovery plans or policy initiatives (Cooke and O'Connor, 2010; Coristine *et al.*, 2014).

To date, perspectives on the field of conservation physiology have focused primarily on the future potential of the discipline (Carey, 2005; Stevenson *et al.*, 2005; Wikelski and Cooke, 2006; Cooke *et al.*, 2013; Madliger and Love, 2015) and have lacked syntheses of past successes and their commonalities (but see Cooke *et al.*, 2014 for a discussion of successes in the integration of behaviour, physiology and conservation). Thus, it appears that the field of conservation physiology may still be largely theoretical. We argue, however, that success stories in conservation physiology are accumulating. Moreover, these successes share commonalities that allow us to delineate themes that characterize the successful application of conservation physiology and highlight where further growth is possible and required.

**Figure 1:**
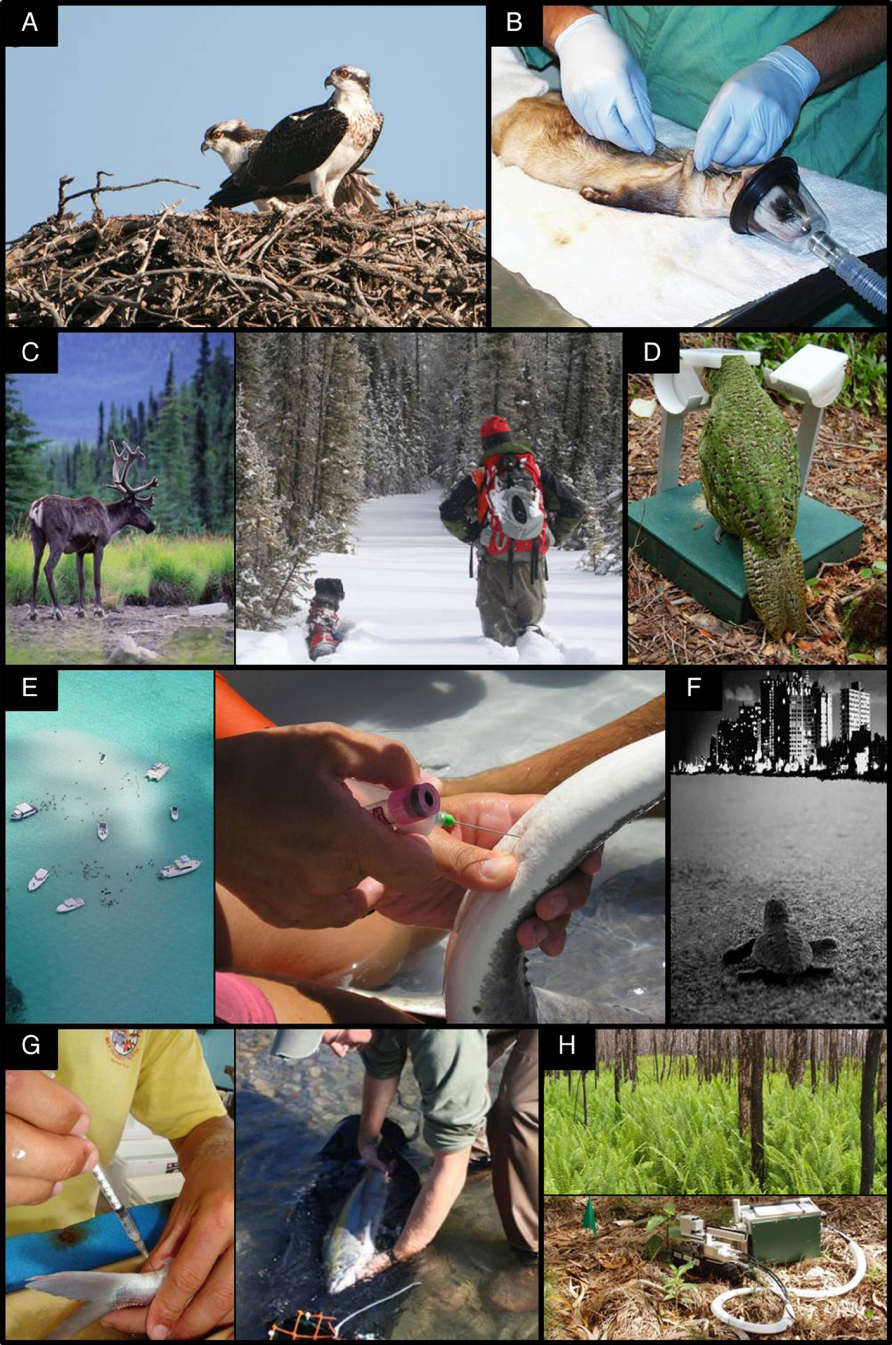
Conservation physiology successes cover a diversity of taxa, ecosystems, landscape scales and physiological systems. For example: (**A**) Birds of prey, such as osprey, have rebounded following regulations on DDT. (**B**) Plague is being combated in the endangered black-footed ferret via a targeted vaccination programme. (**C**) Caribou and wolf populations are being effectively managed via physiological monitoring of scat. In the right photo, a scat detection dog locates samples for subsequent physiological processing. (**D**) Nutrition programmes support successful breeding in the critically endangered kakapo. (**E**) Ecotourism feeding practices are regulated for stingrays in the Cayman Islands. In the right photo, a blood sample is obtained from the underside of the tail to monitor multiple physiological traits. (**F**) Sensory physiology has informed shoreline lighting regulations for nesting sea turtles. (**G**) Physiological monitoring of incidentally-captured fishes can be accomplished through blood sampling (left photo), and recovery chambers have been designed that decrease the stress associated with by-catch in salmonids (right photo). (**H**) Physiological studies have identified native species that tolerate fire caused by exotic species (top panel) and recruit under low light conditions in heavily invaded forests (bottom panel) in Hawaii Volcanoes National Park. Photograph credits: Randy Holland (A); United States Geological Survey National Wildlife Health Center (B); Wayne Sawchuk and Samuel Wasser (C); Kakapo Recovery (D); Christina Semeniuk (E); Sea Turtle Conservancy (F); Cory Suski and Jude Isabella (G); and Jennifer Funk (H).

Here, we posit that conservation physiology has progressed from a nascent, theoretical discipline to an applied one with tangible successes. Specifically, we outline eight diverse topics spanning chemical contamination, integrative wildlife monitoring, nutritional management, disease control, entanglement and collision mediation, control of invasive species, fisheries management and ecotourism, where conservation physiology has resulted in measureable conservation successes. We conservatively define a success as a change in human behaviour, management or policy to the benefit of conservation that has been driven by physiological information. Although we do not provide an exhaustive review, this cross-section highlights the major areas in which conservation physiology has been successful and demonstrates the important role that physiology can play across a broad range of conservation issues (Fig. 1). Finally, we draw on the common features of these successes to identify five emerging themes in the discipline that help to define its current status and breadth. Researchers or managers working within or considering the field of conservation physiology as a framework (see Coristine *et al.*, 2014) for their research activities or management strategies can use this foundation to identify productive pathways forward and foster additional conservation successes.

## Successes in conservation physiology

### Toxicology informs regulatory approaches to environmental chemicals

Environmental toxicology probably represents the longest-standing discipline linking physiological investigations to conservation (Stevenson *et al.*, 2005), with a classic example being that of dichlorodiphenyltrichloroethane (DDT) exposure and biomagnification causing reproductive failure in birds of prey. Specifically, physiologists identified how the breakdown product of DDT inhibits Ca^2+^-ATPase in the shell gland, reducing the deposition of calcium carbonate to the eggshell and resulting in thinner eggshells and reproductive failure (Faroon *et al.*, 2002). These discoveries led to a ban of DDT in many industrialized countries and the consequent recovery of the bald eagle (*Haliaeetus leucocephalus*), brown pelican (*Pelicanus occidentalis*), peregrine falcon (*Falco peregrinus*) and osprey (*Pandion haliaetus*) in North America (Faroon *et al.*, 2002). The DDT success story also spurred the development of other physiological end points used in the ecological risk assessment of chemicals, a process that is ultimately used to assess safety for wildlife (Dickerson *et al.*, 1994). Physiological end points or biomarkers (indicators of a particular disease state or other physiological state of an organism) are now commonly used and range from acetylcholinesterase inhibition to oxidative stress status to immunological indices (Cajaraville *et al.*, 2000; Martin *et al.*, 2010; Beaulieu and Costantini, 2014). In amphibians, the most threatened vertebrate taxon (Stuart *et al.*, 2004; Rohr *et al.*, 2008a), physiological end points such as circulating corticosterone and liver damage have been used as early warning signs of negative effects of fungicide (e.g. chlorothalonil) exposure (McMahon *et al.*, 2011, 2012), and herbicide-induced immunomodulation has been linked with elevated amphibian mortality associated with trematode and chytrid fungal infections (Rohr *et al.*, 2008b,c, 2013). As a result of such research on non-target freshwater vertebrates, regulations on fungicides have been altered to protect susceptible ecosystems better. For example, the Canadian Pest Management Regulatory Agency now requires products containing chlorothalonil to include advisory statements on risk-reduction measures that reduce surface water contamination, such as maintenance of buffer zones between application sites and aquatic areas, and controlled maximal application rates (Health Canada, 2011).

Toxicologists have also demonstrated the mechanisms by which endocrine disruptors cause vertebrate population declines (Vos *et al.*, 2000). Much of this work began when researchers observed male fish producing vitellogenin (a protein normally synthesized by females during oocyte maturation) and eggs in their testes. This feminization was associated with exposure to estrogenic substances, such as synthetic estrogen used in contraceptive pills (Jobling *et al.*, 1996, 1998), and was supported by experimental evidence that synthetic estrogen within the range observed in municipal waste waters can lead to the feminization of males, intersex males, altered oogenesis in females and population declines in fish (Kidd *et al.*, 2007). Likewise, pesticide exposure has been linked to disruption of reproductive and thyroid hormone production, reproductive impairment and disease in amphibians and other vertebrates (Hayes *et al.*, 2006; Rohr *et al.*, 2006; Rohr and McCoy, 2010; Hayes *et al.*, 2011). The clear impacts of estrogens and endocrine disruptors on the sustainability of wild vertebrate populations have encouraged the USA, Japan, European Union and Organization for Economic Co-operation and Development to establish testing approaches and regulatory frameworks to assess and manage the risks associated with chemicals that have endocrine-disrupting potential (Hecker and Hollert, 2011).

### Panels of physiological markers reveal health and stress in wild animals

‘Panels’ are suites of physiological measures (i.e. more than one measure) that provide comprehensive insight into the health and stress status of an individual, and are routinely used in human and veterinary clinical practice (Hindmarsh and Lyon, 1996; Thrall *et al.*, 2012). For example, a wildlife faecal endocrine panel could include glucocorticoids, progestins, androgens and estrogens and sometimes thyroid hormones as well. There is a growing suite of analytical tests, including point-of-care devices that can be used in the field to generate real-time data (Stoot *et al.*, 2014), sophisticated gene expression profiles generated from genomic analyses (e.g. gene arrays, chips) that provide insight on immune function, pathogen presence and metabolic state (Cruz *et al.*, 2012), and novel measures related to oxidative stress (Beaulieu *et al.*, 2013) or telomere length (Lewin *et al.*, 2015; Young *et al.*, 2015). As a result, there is no shortage of tissue-based assays available for assessing the health and physiological status of wildlife.

There have also been major innovations in our ability to collect non-invasive samples from a wide range of species. Given that faeces, urine, hair, feathers, sloughed skin and even respiratory vapour all contain molecules of physiological interest, these samples can be used non-invasively to assess health and stress in wild animals (Hunt *et al.*, 2013; Dantzer *et al.*, 2014). Faecal samples, for example, contain an array of steroid and thyroid hormones, as well as DNA from both prey and host species (Wasser *et al.*, 2010; Vynne *et al.*, 2014). Thus, analysis of faecal hormone titres produces a ‘faecal endocrine panel’ that can provide information on stress physiology (e.g. glucocorticoids and mineralocorticoids), reproductive status (progestins, androgens and estrogens) and nutritional state and metabolic rate (thyroid hormones; Wasser *et al.*, 2011; Ayres *et al.*, 2012; Vynne *et al.*, 2014; Joly *et al.*, 2015). When combined with faecal DNA analyses to confirm species, determine sex and identify individuals, the result is a powerful analytical tool that can identify different environmental stressors and their relative impacts. Indeed, the utility of multiple-measure panels is often in their ability to separate the effects of different stressors to identify the causes of health decline or stress, leading to concrete recommendations. For example, in woodland caribou (*Rangifer tarandus caribou*), a combination of faecal DNA, corticosterone and thyroid measures has helped to delineate the differential impacts of wolf predation vs. human-use patterns associated with oil sands development, leading to a de-emphasis on wolf removal efforts and increased attention to preserving the caribou's access to lichen (Wasser *et al.*, 2011; Joly *et al.*, 2015; personal communication from Dr Samuel Wasser, University of Washington). A similar approach using a panel of faecal reproductive, adrenal and thyroid hormone measures allowed Ayres *et al.* (2012) to compare the impacts of boat traffic and nutritional stress on Puget Sound killer whales (*Orcinus orca*), identifying preservation of the prey base (salmon) as the more important conservation priority.

Beyond faecal hormones, -omics tools (including transcriptomics, proteomics and genomics) are increasingly being applied to conservation problems, enabling the rapid screening of thousands of genes related to physiological and biochemical end points, such as immune function and metabolic state. For example, Miller *et al.* (2011) took minimally invasive gill biopsies from migrating sockeye salmon (*Oncorhynchus nerka*) that were released with telemetry transmitters, enabling researchers to identify physiological signatures associated with failed migrants. Transcriptomics has also been used for environmental screening of condition, immunity and stress in steelhead (*Oncorhynchus mykiss*) on the Columbia River (Connon *et al.*, 2012). These tools have helped to identify suites of factors that are associated with environmental stressors and disease in wild salmonids, thus improving management actions by allowing practitioners to refine and justify harvest restrictions in Canada, leading to a greater balance among different stakeholder groups (Cooke *et al.*, 2012). It is anticipated that as more multipanel assessments become part of long-term routine monitoring, it will be possible to develop mechanistic models to determine better how human activities influence a multitude of animal populations.

### Nutritional physiology improves management of captive and wild populations

The physiology underlying the nutritional needs of animals has been well explored in the context of agriculture (McDonald *et al.*, 2002) and zoos (Dierenfeld, 1997), and—particularly for mammals—there are well-established markers available to allow assessment of nutritional health (e.g. Underwood, 1977; McDowell, 1989). In the context of conservation, nutritional physiology is particularly important in captive rearing programmes, in captive rearing for release programmes and in heavily managed populations, where food supplements may be provided to avoid disease and improve performance (Tracy *et al.*, 2006). Specifically, captive populations are a critical component of final-stage species conservation and have been somewhat successful for recovering critically endangered species and for supplementing populations (Philippart, 1995; Snyder *et al.*, 1996). Captive animal nutrition (including captivity for conservation purposes) is often developed through trial and error, combining field observations with *ad hoc* choice experiments, with reference to existing captive diets or nutritional information for related laboratory model species (Dierenfeld, 1997). Although health and performance provide the most appropriate measure of success, sometimes simply identifying suitable food can be a challenge (e.g. Honan, 2008). In other cases, physiological studies can be used to simplify captive diets. For example, tuatara (*Sphenodon* spp.), reptiles endemic to New Zealand, have been observed to eat seabird chicks in the wild, resulting in free-living individuals having high plasma levels of polyunsaturated fatty acids (Cartland *et al.*, 1994; Cartland-Shaw *et al.*, 1998). Although dietary supplementation with fish oil modified the plasma composition, this did not affect growth rate, metabolic rate or survivorship, requiring no change to the diet in captivity (Blair *et al.*, 2000), and no specific changes to captive diets were made (personal communication from Dr Alison Cree, University of Otago).

Nutrition-based diseases may be avoided by the provision of micronutrients, and often the underlying cause of such diseases can be detected only via a combination of physiology and pathology. For example, threatened black stilts (kāki, *Himantopus novaezelandiae*) are captive reared for release in New Zealand's South Island, but there was initially considerable variation in hatching mortality between eggs collected from the wild (<15%) and those derived from captive birds (>50% perihatching mortality). Although the diet contained sufficient iodine for domestic poultry, increased incidence of goitres and low thyroxine titres in captive vs. wild birds led to a hypothesis of iodine deficiency. Supplementation of dietary iodine in the entire captive population increased serum thyroxine levels and led to consistently low perihatching mortality (Sancha *et al.*, 2004), and remains part of the captive diet (personal communication from Dr Richard Maloney, New Zealand Department of Conservation).

Nutritional physiology can also inform management decisions in wild and semi-wild populations at both the individual and landscape scales. For example, Bryant (2006) used doubly (isotopically) labelled water to estimate the energy expenditure in both free-ranging and captive kakapo (*Strigops habroptilus*). Mass-corrected estimates of energy expenditure are used specifically to determine the supplementary feeding protocol for this critically endangered parrot. As the diet of both males and females is supplemented to achieve a threshold minimal mass for breeding, these data on energy expenditure allow managers to regulate the mass of birds in the approach to the breeding season to prevent females from crossing an upper threshold at which offspring become male-biased (personal communication from Daryl Eason, New Zealand Department of Conservation). At the landscape scale, understanding the physiology underlying threatened desert tortoise (*Gopherus agassizii*) nutritional requirements (Tracy *et al.*, 2006) has determined management decisions regarding habitat quality (US Fish and Wildlife Service, 2011).

Finally, nutritional physiology can identify sublethal impacts that can be traced back to large-scale ecosystem processes that, in some instances, have informed intervention. For example, the Laurentian Great Lakes have experienced widespread changes in food web structure owing to overexploitation, changes in habitat quality and introduction of non-native species (Mills *et al.*, 1994). Native lake trout (*Salvelinus namaycush*) populations have experienced dramatic population declines, which are partly attributed to thiamine (vitamin B_1_) deficiency arising from a switch to consumption of non-native alewife (*Alosa pseudoharengus*), which contain high levels of thiaminase that breaks down thiamine (Brown *et al.*, 2005). After this problem was identified (Krueger *et al.*, 1995), fisheries managers were able to reduce populations of alewife in efforts to restore native lake trout populations, which has been somewhat successful as part of a multifaceted native restoration plan (Dettmers *et al.*, 2012).

### Principles of ecological immunology aid in disease control

An understanding of the physiological function of immune systems, and acquired immune mechanisms specifically, has been instrumental to the development of successful vaccination campaigns with dramatic conservation implications. The key precursor of a successful vaccination programme for a host species of conservation concern is demonstrating that the host has the physiological capability to acquire immunity upon exposure to either dead or attenuated pathogen, and that this enhanced immunity is greater than any immunosuppressive effects of the pathogen. As an example, amphibians are experiencing widespread population declines and extinctions associated with chytrid fungal infections (Rohr and Raffel, 2010; Raffel *et al.*, 2013; Venesky *et al.*, 2014). Recent work revealed that repeated exposures of amphibians to chytrid increased lymphocyte abundance in hosts and lymphocyte proliferation when cultured with the dead pathogen. Moreover, immune memory stimulated by exposure to dead chytrid exceeded the immunosuppression caused by the fungus, resulting in reduced chytrid loads and enhanced frog survival (McMahon *et al.*, 2014).

The concept of induced adaptive immunity has been applied successfully to rescue several host species that experienced declines from introduced pathogens. For example, the morbillivirus that causes rinderpest was introduced into northwestern Africa in the 1880s and resulted in 90% mortality of domestic and wild ungulates (Mariner *et al.*, 2012) and subsequent declines of their canid and felid predators (Dobson *et al.*, 2011). A thermostable vaccine was administered to domestic livestock throughout Africa and resulted in an elimination of the disease from wild ungulates and a subsequent surge in lion and hyena populations (Dobson *et al.*, 2011). When rabies outbreaks threatened the world's rarest canid, the Ethiopian wolf (Randall *et al*., 2006), managers implemented a baited oral vaccination campaign focused at the corridor between an outbreak and susceptible wolf subpopulations and successfully prevented incursion of the epidemic into the vaccination zone (Haydon *et al.*, 2006). Likewise, plague caused by the introduced bacterium *Yersinia pestis* is considered a factor in the declines of prairie dogs and the black-footed ferret, possibly the most endangered mammal in North America. Laboratory studies revealed that a vaccine conferred protection against *Y. pestis*, and agencies have now widely distributed vaccine-laden bait and are tracking the recovery of prairie dog and ferret populations (USGS, 2011). Finally, the discovery of persistence of maternal antibodies in chicks of a long-lived colonial seabird species, the Cory's shearwater (*Calonectris borealis*), has influenced the design of vaccination programmes to protect nestlings of Procelariiforms (shearwaters, albatrosses and petrels) against recurrent epizootics in breeding colonies (Garnier *et al.*, 2012). Specifically, female albatross species threatened by avian cholera (*Pasteurella multocida*) on Amsterdam Island, southern Indian Ocean are being vaccinated to allow transmission of persisting maternal antibodies to their chicks over several breeding attempts (Weimerskirch, 2004; Garnier *et al.*, 2012; Ramos *et al.*, 2014; personal communication from Dr Thierry Boulinier, Université Montpellier). In summary, these examples emphasize the value of understanding immunology in a physiological context and, subsequently, implementing vaccines to manage threatened host species over vast geographical regions.

### Sensory-based conservation strategies mitigate human–wildlife conflicts

Sensory physiology has guided conservation management in diverse scenarios, dictating strategies that exploit sensory modalities either to attract animals to desirable locations or to deter them from undesirable ones (Cooke *et al.*, 2013). Environmental alterations resulting from anthropogenic activities can create novel sensory cues (visual, auditory, olfactory, etc.) that mimic naturally occurring signals (Robertson *et al.*, 2013) or create features that are not easily detectable and can lead to collisions or entanglement (Martin and Crawford, 2015). Sensory-based interferences have been documented in relationship to a variety of structures and objects, such as light sources (Gaston *et al.*, 2012), fishing nets and lines (Southwood *et al.*, 2008), marine debris (Horváth *et al.*, 2009), wind turbines (Kuvlesky *et al.*, 2007), windows (Klem, 2009), power lines (Alonso *et al.*, 1994) and reflective solar panels (Horváth *et al.*, 2009). Overall, the associated negative consequences for wildlife of such sensory traps often manifest as suboptimal choices of habitat, mates, migration routes or food and, in some cases, death (Schlaepfer *et al.*, 2002).

A consideration of sensory physiology has allowed managers and industries to alter structures and equipment to minimize influences on wildlife by tailoring aversion measures to the sensory capacities of targeted wildlife (Madliger, 2012; Martin and Crawford, 2015). Specifically, the measurement of visual and auditory sensitivities has pinpointed the most effective strategies for deterring wildlife. This approach has been particularly successful in the fishery sector, where acoustic alarms have been designed to take advantage of the auditory sensitivities of aquatic mammals, subsequently reducing incidental captures (i.e. by-catch; Cox *et al.*, 2007). This is critical to conservation because estimates of total global by-catch are as high as 38.5 million tonnes per year (Davies *et al.*, 2009) and thus, mitigation measures based on targeted sensory approaches can have far-reaching implications for wildlife incidentally influenced by fishing practices. For example, ‘pingers’, which create continuous bursts of sound, have been implemented in the US Northeast gillnet fishery and have reduced harbour porpoise (*Phocoena phocoena*) by-catch rates by 50–70% (Palka *et al.*, 2008). Likewise, in the California drift gill net fishery, pingers reduced beaked whale (*Ziphiidae* spp.) by-catch to zero (Carretta *et al.*, 2008) and have significantly decreased incidental captures of short-beaked common dolphins (*Delphinus delphis*) and California sea lions (*Zalopbus californianus*; Barlow and Cameron, 2003). In the context of hydropower facilities, a host of stimuli, such as strobe lights, high-intensity sound and bubble curtains (tactile deterrents) have been used to prevent impingement or entrainment of fishes (Noatch and Suski, 2012). Other acoustic alarms that use low frequencies and harmonics have also reduced whale collisions with cod (*Gadus morhua*) and capelin (*Mallotus villosus*) fishing gear in Newfoundland, Canada (Lien *et al.*, 1989). Beyond noise-based mitigation measures, there is extensive research into gear modifications that can exploit other sensory systems through olfactory, visual and chemosensory cues to decrease incidental captures in cetacean, avian, sea turtle and elasmobranch species (Brothers *et al.*, 1999; Pierre and Norden, 2006; Wang *et al.*, 2010; Chosid *et al.*, 2012; Jordan *et al.*, 2013).

A sensory-based approach has also been applied to the problem of avian collisions with buildings. Birds are particularly vulnerable to collisions with human structures because their high-resolution vision is limited to the lateral view, lighted buildings can act as an attractant (Martin, 2011), and they are unable to distinguish the reflection of vegetation in mirrored surfaces from natural features (Klem *et al.*, 2009). In the USA alone, annual mortality caused by building collisions is estimated to be between 365 and 988 million birds (Loss *et al.*, 2014), and collisions are thought to represent the second largest cause of anthropogenically linked mortality in birds worldwide (Klem, 2009). However, a number of approaches based on knowledge of the visual perception of birds can reduce window collisions by nearly 60% (Klem and Saenger, 2013). Fritted (patterned) glass, uniformly spaced decals and ultraviolet-absorbing and -reflecting films targeted to wavelengths visible to birds effectively reduce avian collisions with buildings (Klem, 2009; Klem and Saenger, 2013). Importantly, many major cities, including Toronto, Vancouver, Chicago, New York City and San Francisco, are incorporating bird-friendly, sensory-based guidelines into legislation, development plans and ‘lights out’ awareness programmes to minimize mortality of avian species caused by building collisions, particularly during migratory periods (City of Chicago, 2007; City of Toronto, 2007; New York City Audubon Society, 2007; San Francisco Planning Commission, 2011; City of Vancouver, 2015). In addition, larger federal programmes in the USA, such as Leadership in Energy and Environmental Design (LEED), which provides certification for green buildings, have also begun to provide credits for building designs that include high-visibility facades for bird-collision reduction (US Green Building Council, 2015). In another vision-based conservation strategy, federally listed sea turtle hatchlings that are disoriented by shoreline lighting have benefited from regulations in Florida and South Carolina aimed at altering the intensity and wavelengths of light sources based on the visual sensitivities of affected species (Lohmann *et al.*, 1997; Salmon, 2006).

### Physiological knowledge aids in control of invasive species and subsequent restoration

Invasive species are considered to be a leading cause of animal extinctions worldwide (Clavero and Garcia-Berthou, 2005) and can have many complex and often negative ecological and evolutionary impacts across taxa (Vitousek *et al.*, 1997; Wilcove *et al.*, 1998; Pimentel *et al.*, 2000; Vilá *et al.*, 2011). Although the concept of conservation physiology has been applied most directly to the study of native species threatened by environmental change, it has also aided in the identification of physiological traits of invasive species that can be harnessed to direct control and mitigation efforts and to predict further spread (Funk *et al.*, 2008; Funk, 2013; Lennox *et al.*, 2015). Specifically, the application of physiology to combat invasive species typically identifies traits that impact whole-organism function, such as metabolism, nutritional status or thermal tolerance (Chown, 2012). In this way, physiology can be used to determine management approaches that may best exploit a given trait, which weakens or eliminates the capacity of a species to invade a non-native habitat. For example, the thermal tolerances of wood-boring insects [e.g. Asian longhorned beetle (*Anoplophora glabripennis*)] have been used to determine the minimal heat treatments required by the International Plant Protection Convention for phytosanitary treatment of wood packaging material (e.g. pallets and crates). The associated international phytosanitary standards (IPPC, 2009), which allow for heat or fumigation as control measures, are estimated to have decreased infestation rates of wood and bark pests by 36–52% worldwide (Haack *et al.*, 2014). This reduction in infestation will decrease propagule pressure (i.e. the number of viable insects entering a new location) and therefore the likelihood of subsequent invasion (Brockerhoff *et al.*, 2014).

In other cases, a consideration of physiology has contributed to the control of invasive species in already-established locations by decreasing their ability to function or survive. For example, sea lamprey (*Petromyzon marinus*), which parasitize adult top-predator fish during the juvenile stage, colonized the Laurentian Great Lakes during the late 1900s and caused severe losses to sport and economically important fishes (Chapman and Bolen, 2015). Research conducted in the 1960s–1970s indicated that application of the chemical 3-trifluoromethyl-4-nitrophenol (TFM) inhibited ATP production and mitochondrial oxidative phosphorylation, thus shutting down aerobic respiration and causing mortality, while posing minimal health risks for other wildlife or humans (Menzie and Hunn, 1976; Hubert, 2003). According to the Great Lakes Fishery Commission (2015), the application of TFM, along with building barriers and trapping, has been a ‘remarkable success’, because it has reduced sea lamprey populations by 90% in most areas of the Great Lakes.

A consideration of physiology has also refined management decisions involving use biological control agents. For example, the *Tamarix* leaf beetle, *Diorhabda* spp. (Chrysomelidae), was released in the USA to control *Tamarix* (DeLoach *et al*., 2003), an invasive tree/shrub that has negatively impacted biodiversity, water resources and ecosystems functions in arid and semi-arid riparian ecosystems of the western USA and Northern Mexico (Shafroth *et al.*, 2005). Ongoing research has identified geographical gradients in plant tolerance to herbivory, such that *Tamarix* populations from warmer climates are more susceptible to defoliation by *Diorhabda* than populations from cooler climates (Williams *et al.*, 2014). Gradients in herbivory tolerance appear to be related to specific physiological traits, such as the allocation of recent photosynthates to growth and labile carbon storage, which may make *Tamarix* genotypes in some regions more susceptible to biocontrol than others (Hultine *et al.*, 2015). Specifically, riparian restoration priorities are currently being targeted in Arizona based on the identification of *Tamarix* carbon allocation strategies across broad, macrophysiological scales (Orr *et al.*, 2014).

A consideration of the physiological differences among native and invasive plant species can also directly improve management practices (e.g. Funk *et al.*, 2008; Funk, 2013). Physiological studies of light and fire tolerance have impacted management protocols in Hawaii Volcanoes National Park (HAVO), where managers are tasked with conserving large tracts of native forests that are threatened by invasive species. For example, shade-intolerant invasive grasses suppress the recruitment of native ferns and woody canopy species in mesic forests. Funk and McDaniel (2010) manipulated light levels in a disturbed forest and assessed species differences in photosynthetic rate, growth and survival. They concluded that lowering light levels by establishing canopy species may suppress the growth of invasive grasses with no adverse effects on native woody species. The study identified several fast-growing native species ideal for restoration, and the resultant planting palette has been applied to restoration of 12–16 hectares. Understanding the effect of light on seedling emergence and growth has also shaped how HAVO managers restore forests in the presence of a woody canopy invader. Girdling invasive fire tree (*Morella faya*) was found to be more effective in promoting native species than logging trees (Loh and Daehler, 2007, 2008). Logging increased light levels, which promoted invasion by fast-growing shade-intolerant exotic species, whereas the slow death of fire tree by girdling allowed establishment of native plants accustomed to partial shade. During the 1960s, the invasion of fire-adapted invasive grasses increased fire frequency 3-fold in seasonally dry woodland in HAVO. Given that these grasses are impossible to eradicate, fire will continue to hinder restoration efforts. When planting native species, managers now eschew previously dominant but fire-sensitive species (e.g. *Metrosideros polymorpha*) for fire-adapted native species. Studies of fire tolerance and colonization potential after fire led to plant palettes for several large-scale restoration efforts in the park (Loh *et al.*, 2007, 2009; McDaniel *et al.*, 2008).

### Fisheries management is improved through physiological monitoring

Inland and marine fisheries resources are globally important as a food supply and are culturally and economically important in many places for recreation. When coupled with anthropogenic stressors, such as habitat destruction or alteration (e.g. construction of dams, land use change), pollution and climate change, a diversity of fishes have been experiencing population declines at both a local and global scale. Despite decades of regulation and oversight, many marine fishery stocks are currently being fully exploited or overexploited (FAO, 2012), while globally, freshwater fishes are among the most threatened taxa on the planet (Ricciardi and Rasmussen, 1999; Dudgeon *et al.*, 2006). This decline in abundance and richness speaks to the need for the development of novel tools and technologies to monitor the health of animals and provide effective mitigation strategies to maintain populations.

Studies related to the conservation of Pacific salmon (*Oncorhynchus* spp.) represent one of the most celebrated and relevant models of using animal physiology to achieve conservation success. Historically, Pacific salmon were abundant on the west coast of North America and provided a host of critical ecosystem services ranging from a food source for humans to delivering nutrients to terrestrial ecosystems to cultural value (Janetski *et al.*, 2009; Hocking and Reynolds, 2011). Owing to logging, dams, irrigation, commercial and sport fisheries, as well as increased human populations, Pacific salmon numbers have declined precipitously in the past century, with a number of species and stocks throughout their range currently listed as threatened or endangered (Gresh *et al.*, 2000; Ford, 2011; Quinones *et al.*, 2014). Farrell *et al.* (2001a) used physiological response variables to demonstrate that towing non-target Coho salmon (*Oncorhynchus kisuitch*) captured as by-catch in commercial nets promotes physiological recovery and increased post-release survival, even for fish that appeared moribund at the time of capture in gill nets (Farrell *et al.*, 2001a,b), leading to regulations requiring gill net boats to have recovery boxes attached to vessels to facilitate recovery of coho by-catch. Likwise, Donaldson *et al.* (2013) demonstrated that comparative physiology and radio-telemetry could be combined with human dimensions surveys to address revival strategies for angled and released sockeye salmon (*Oncorhynchus nerka*), leading to public outreach activities intended to improve handling of fish that are to be released. In addition, Young *et al.* (2006) identified biomarkers correlating with physiological performance that could be used to predict whether individual fish were likely to reach spawning grounds compared with those that did not continue migrations, providing managers with a tool to identify instances where escapement targets may not be met because of en route mortality. Together, these studies, as well as others (e.g. Cooke *et al.*, 2012), demonstrate how integrating physiological tools into biological problems can achieve conservation success for an economically and ecologically important group of fish species.

The recent development of metrics to assess the whole-animal response to capture stressors has also provided fisheries managers with a simple yet effective method for defining capture stress and improving conservation activities. A number of fish species are captured by either recreational or commercial harvesters and are subsequently released, owing to regulations mandating release (i.e. time of year, size) or a voluntary conservation-based ‘catch-and-release’ ethic (Davis, 2002; Arlinghaus *et al.*, 2007). However, during a capture event, fish can experience a range of different stressors, such as depth change, exercise, crowding and handling, all of which can lead to elevated levels of physiological stress (Farrell *et al.*, 2001b; Suski *et al.*, 2003). In extreme cases, the stress and disturbance related to capture can cause mortality, which can negate efforts to release captured individuals successfully and can translate to negative population-level changes (Davis, 2002). Davis (2010) showed that fish have a number of ecologically relevant, involuntary reflex responses that are correlated positively with the magnitude of a physiological stressor. As such, these reflex indices can be collected easily and rapidly in the field from a range of fish species, and subsequently, used to predict disturbance level and subsequent mortality using a process called Reflex Action Mortality Predictor (RAMP; Davis, 2007). For example, Raby *et al.* (2012) showed that RAMP scores, collected as part of a fishery mandating the release of non-target coho salmon (*Oncorhynchus kisutch*), were able to predict both mortality and behaviour of wild fish after release. As a result, the RAMP procedure provides a simple, inexpensive and effective protocol to collect data on fisheries mortality rates quickly and easily in the field that has been ground-truthed in relationship to physiological parameters and provides information on how changes to fishery practices can translate to improved survival for released individuals.

### Monitoring of energetics and stress refines ecotourism practices

Ecotourism refers to a sector of the tourism industry that is nature based, rooted in environmental education and sustainably managed (Blamey, 2001) and, ideally, represents an opportunity to promote the conservation of ecosystems or species of interest while achieving economic benefits (Ellenberg *et al.*, 2006). However, many of the activities associated with ecotourism can lead to disturbances in the behaviour, reproduction and persistence of terrestrial and aquatic wildlife (Newsome *et al.*, 2005). Measures of physiological traits have allowed for the relatively rapid assessment of these effects in a diversity of wildlife and, most importantly, in many instances have enabled researchers to make management recommendations that can reduce the associated impacts on sensitive populations.

A particularly strong example of the power of physiological measures for the assessment of effects and subsequent refinement of the ecotourism industry focused on southern stingrays (*Dasyatis americana*). This species is the basis of a feeding attraction at ‘Stingray City Sandbar’ (SCS) in the Cayman Islands that brings in more than 1 million tourists annually. Stingrays at SCS are part of a wild population, but can be subjected to up to 2500 tourists simultaneously (from up to 40 boats) participating in diving, snorkelling, touching and feedings (Semeniuk *et al.*, 2007). By comparing stingrays inhabiting tourist sites and non-visited sites, Semeniuk *et al.* (2009) showed that animals exposed to ecotourism had lower haematocrit, lower total serum protein concentrations and reduced antioxidant capacity, indicating negative physiological consequences of tourism operations. In addition, fatty acid profiles of stingrays fed the non-natural diet associated with tourism activities did not obtain a nutritional lipid composition comparable to prey eaten in the wild, with potential consequences for growth, immune function, parasite and disease prevalence, and ultimately, survival (Semeniuk *et al.*, 2009). Based on tourist surveys and the predicted health effects from the physiological studies, Semeniuk *et al.* (2010) then developed an integrated system dynamics model for the management of tourist–stingray interactions at SCS, which predicted the state of the tourism attraction over time in relationship to stingray population size, life expectancy and tourist visitation under various management scenarios. These findings allowed for management recommendations directly to Caymanian stakeholders that included decreasing the amount of artificial food to promote natural foraging, changing the composition of supplemented food, continued monitoring of fatty acid levels as a bioindicator, limiting total numbers of boats and people to eliminate crowding, and expanding tourism sites (Semeniuk *et al.*, 2007, 2009, 2010; Semeniuk and Rothley, 2008). Overall, this approach enabled regulators to choose management plans that would ensure tourist satisfaction and continued visitation despite stricter regulations that benefit wildlife (personal communication from Dr Christina Semeniuk, University of Windsor). Taken together, these studies have inspired a call for change in policies for recreational marine ecotourism to minimize the impacts on population health for rays in other areas, such as the Mediterranean, Southeast Asia and Africa (Lloret, 2010; Corcoran *et al.*, 2013; Ward-Paige *et al.*, 2013), as well as for other marine fishes (e.g. Hammerschlag *et al.*, 2012).

Many other species targeted specifically by or indirectly exposed to the tourism industry have also been assessed using diverse physiological tools. For example, endangered yellow-eyed penguins (*Megadyptes antipodes*; Ellenberg *et al.*, 2007), juvenile hoatzins (*Opisthocomus hoazin*; Müllner *et al.*, 2004) and Western capercaillie (*Tetrao urogallus*; Thiel *et al.*, 2008) in areas with tourism exposure show higher levels of glucocorticoids (i.e. stress hormones) than individuals in undisturbed sites. In many cases, glucocorticoid levels and heart rate telemetry metrics have correlated with reproductive and/or survival parameters that justify regulation of tourism activities based on life-history stage, location and intensity (i.e. distance) for avian species (Müllner *et al.*, 2004; Ellenberg *et al.*, 2006, 2007). In particular, this type of work in yellow-eyed penguins (Ellenberg *et al.*, 2006, 2007), one of the world's rarest penguin species, has improved visitor information panels and viewing hides for tourists, and breeding areas are routinely closed to access during the breeding season (personal communication from Dr Ursula Ellenberg, La Trobe University). In addition, at a viewing site where visitors must walk along the beach to access viewing hides (Sandfly Bay, New Zealand), a volunteer warden programme has been coordinated by the New Zealand Department of Conservation to keep visitors out of breeding areas and to reduce disruption of penguin landing (personal communication from Dr Ursula Ellenberg, La Trobe University). Overall, the measurement of physiology has provided robust biomarkers of condition and disturbance level that can refine ecotourism activities to minimize impacts on wildlife.

## Emerging themes and conclusions

### Conservation physiology goes beyond documenting change

The success stories we have outlined indicate that conservation physiology is, in many cases, fulfilling the goal outlined in its most recent definition, which places specific emphasis on ‘solving conservation problems across the broad range of taxa’ (Cooke *et al.*, 2013). In addition to identifying impacts of disturbance or environmental change, physiology has allowed managers to delineate and prioritize mitigation strategies, often because physiology provides mechanistic insight into the causes of change (Carey, 2005; Wikelski and Cooke, 2006). As a result, conservation physiology has allowed for targeted strategies that can: (i) limit anthropogenic activities in space, time or intensity (e.g. yellow-eyed penguin ecotourism); (ii) focus strategies to target certain life-history stages or aspects of ecology/habitat (e.g. control of invasive sea lamprey); (iii) control the spread of disease (e.g. rinderpest eradication in Africa); and (iv) alter human structures and activities to limit influences on wildlife (e.g. window redesign to limit bird strikes). Moving forward, we propose that conservation physiology be viewed more strongly as a set of tools for addressing, rather than merely documenting, conservation issues.

### The tools available and contributing to the field are more diverse than glucocorticoids

Although measurements of stress hormones (i.e. glucocorticoids) dominate the conservation physiology literature for vertebrates (Lennox and Cooke, 2014), the successes we have identified are varied and rely on diverse physiological traits related to immunity, nutrition, toxicology, sensory physiology, oxidative status, haematology, metabolism and reproduction. Thus, rather than defaulting to the measurement of stress hormones, which are often highly context dependent and difficult to interpret (Breuner *et al.*, 2008; Bonier *et al.*, 2009; Madliger and Love, 2014), conservation physiologists should incorporate additional measures into their panels. Using physiological measures that provide meaningful information, rather than assuming that any disturbance will be reflected unambiguously in stress levels, will push conservation physiology further towards the diverse discipline it has been proposed to be (Wikelski and Cooke, 2006; Cooke *et al.*, 2013), in terms of both on-the-ground conservation and the accumulation of a literature base that can benefit evidence-based conservation.

### Conservation physiology approaches can be transferable among species, locations and times

In our experience, physiological approaches to conservation are sometimes criticized for being species, site or time specific, thereby limiting the general utility of the solutions. Although in some cases management strategies may be very specialized, the outlined successes indicate that conservation physiology has not suffered from a lack of transferability in many areas. For example, toxicological research on pesticides and other endocrine-disrupting chemicals has had far-reaching conservation implications for birds of prey and aquatic wildlife worldwide. Likewise, sensory physiology work that has helped to identify window designs that prevent bird strikes has benefited hundreds of species of migratory songbirds in cities throughout North America, and vaccination campaigns, such as the targeted programme for rinderpest, have eradicated disease from multiple ungulate species across entire continents. Importantly, conservation physiology approaches are contributing to both reactive conservation, such as the problem solving associated with sensory interferences, disease epidemics, ecotourism and fisheries by-catch, and proactive conservation, such as the modelling of invasive species spread, biological control of invasive species, health and reproductive monitoring, and forecasting of how organisms will respond to climate change or other environmental alterations. Finally, the knowledge gained through general studies in physiological ecology and evolutionary physiology continues to inform the rapid development of tools for conservation physiology, and many more opportunities are available to advance this development further (Madliger and Love, 2015).

### Highly targeted solutions can allow for human use while simultaneously benefiting imperiled populations

Given that physiology can impart the ability to pinpoint the mechanism behind a conservation issue (Carey, 2005), techniques can often be highly targeted to accomplish conservation goals in the most parsimonious way possible. As a result, many solutions based on physiological knowledge have allowed human use or activity to continue to occur, while benefiting or ameliorating conflicts for wildlife. For example, recovery techniques, harvesting regulations and deterrents used in the fishery sector have initiated strategies that simultaneously allow harvest and maintenance of wild populations of commercially important fish species while minimizing impacts to non-target species. The sensory-based modifications to windows and shoreline lighting that have benefited migratory birds and endangered sea turtles, respectively, continue to allow for building facades and lighting of structures to maintain aesthetic and human use. Finally, the physiological knowledge gained from studies in the ecotourism industry has refined practices so that tourist visitation can continue while minimizing negative influences on wildlife such as yellow-eyed penguins and stingrays. Overall, the incorporation of physiology has provided concrete evidence for how and why conservation strategies are necessary, allowing for justification of strategies, maintenance of stakeholder relationships and beneficial changes for humans and wildlife.

### Evidence of success can be difficult to find in primary literature, but it is gradually and continuously occurring

A repeated lesson across the above studies has been that, although policy changes can often be slow and incremental, change occurs if very clear recommendations are persistently brought to managers, mass media and/or policy-makers. As with any conservation endeavour, changes in human behaviour, management or policy can take time because of logistical, monetary and dissemination constraints. As a result, the identification of success stories where physiological work led to downstream management effects often required the piecing together of multiple, sometimes disparate, studies. In many cases, conservation results were not easily accessible through searches of the primary literature and required direct communication with researchers or practitioners, or searches of government websites and other documents. However, conservation physiology has been accumulating success stories prior to its formal description as a discipline, and we argue that it is keeping pace with other more recent subfields of conservation biology, such as conservation behaviour. Overall, we support a recent suggestion by Cooke (2014) that the single biggest challenge for conservation physiology is to ensure that findings are relevant to practitioners (Cooke and O'Connor, 2010), but we advocate that the highlighted successes provide optimism regarding our ability to overcome this impediment.

### Conclusion: conservation physiology is progressing past theoretical and proposed applications

The potential applications of a physiological approach to conservation are well established (Carey, 2005; Wikelski and Cooke, 2006; Cooke *et al.*, 2013), and a theoretical framework has recently been proposed to guide progression of the field further by defining information flows within and between science and policy-makers (Coristine *et al.*, 2014). Although many of the concrete successes in the field have occurred in animal systems, the potential for success in plants is clear and also gaining momentum. Although a recent bibliographic analysis concluded that, from a publication perspective, the overall pace of integration between conservation and physiology has been slower than the opportunities would potentially warrant (Lennox and Cooke, 2014), the concerted summary of successes provided here indicates that conservation and physiology have been well integrated in diverse, far-reaching and beneficial ways that may not be readily apparent from a standardized literature search. Moving forward, further success will be fostered by linking individual-level physiological traits with population- and species-level phenomena (Cooke and O'Connor, 2010; Cooke *et al.*, 2013). In addition, many successful strategies have come and will continue to be developed from merging multiple approaches with conservation and physiology, such as behaviour, genetics, social science and medicine. In this way, conservation physiology is becoming a body of work that is not defined by one type of approach, physiological measure, taxa or conservation issue, but by the diversity it encompasses. Most importantly, the success stories discussed here illustrate that physiological knowledge continues to have the potential to make considerable contributions to conservation, that it has been doing so for decades and that it will continue to make broad strides during a time when its diversity should be seen as an enormous benefit to global conservation goals (Tallis and Lubchenco, 2014).

## Funding

This work was supported by the Society for Integrative and Comparative Biology; the University of Windsor, Ontario, Canada; Dalhousie University, Nova Scotia, Canada; and the Canadian Society of Zoologists. C.L.M. was supported by a Natural Sciences and Engineering Research Council of Canada PGS-D (427552). S.J.C. and O.P.L. are supported by the Canada Research Chairs program. E.J.C. was supported by a grant from the National Science Foundation (BCS-1134687). K.R.H. was supported by grants from the National Science Foundation's MacroSystems Biology program (award no. 1340856) and the US Department of Agriculture (NRI 2015-67013-23138). J.R.R. was supported by grants from the National Science Foundation (EF-1241889), National Institutes of Health (R01GM109499, R01TW010286), US Department of Agriculture (NRI 2006-01370, 2009-35102-0543) and US Environmental Protection Agency (CAREER 83518801).
